# Vestibular Implant Surgery: How to Deal With Obstructed Semicircular Canals—A Diagnostic and Surgical Guide

**DOI:** 10.1177/19160216241291809

**Published:** 2025-01-01

**Authors:** Raymond van de Berg, Joost Johannes Antonius Stultiens, Marc van Hoof, Vincent Van Rompaey, Janke Roelofke Hof, Bernd Lode Vermorken, Benjamin Volpe, Elke Maria Johanna Devocht, Angélica Pérez Fornos, Alida Annechien Postma, Vincent Lenoir, Minerva Becker, Nils Guinand

**Affiliations:** 1 Department of Otorhinolaryngology—Head and Neck Surgery, School for Mental Health and Neuroscience, Faculty of Health Medicine and Life Sciences, Maastricht University Medical Center, Maastricht, The Netherlands; 2 Department of Otorhinolaryngology — Head and Neck Surgery, Antwerp University Hospital, Department of Translational Neurosciences, Faculty of Medicine and Health Sciences, University of Antwerp, Antwerp, Belgium; 3 Division of Otorhinolaryngology—Head and Neck Surgery, Department of Clinical Neurosciences, Geneva University Hospitals, University of Geneva, Geneva, Switzerland; 4 Department of Radiology and Nuclear Medicine, School for Mental Health and Neuroscience, Faculty of Health Medicine and Life Sciences, Maastricht University Medical Center, Maastricht, The Netherlands; 5 Division of Radiology, Diagnostic Department, Geneva University Hospitals, University of Geneva, Geneva, Switzerland

**Keywords:** vestibular function, vestibular implant, vestibular implantation, vestibular prosthesis, implanted electrodes, semicircular canals, extracellular matrix proteins, fibrosis, ossification, magnetic resonance imaging, computed tomography, surgical procedures

## Abstract

**Background:**

A vestibular implant can partially restore vestibular function by providing motion information through implanted electrodes. During vestibular implantation, various obstructions of the semicircular canals, such as protein deposits, fibrosis, and ossification, can be encountered. The objective was to explore the relationship between preoperative imaging and intraoperative findings of semicircular canal obstruction and to develop surgical strategies for dealing with obstructions of the semicircular canal(s) in patients eligible for vestibular implantation.

**Methods:**

Patients undergoing vestibulocochlear implantation (in an active clinical trial) were included in the current study when preoperative imaging indicated an obstruction in the semicircular canal. Preoperative imaging consisted of CT and MRI scans. During surgery, the bony semicircular canals were skeletonized (“bluelined”) to identify the course of the canals and create a fenestration to insert the electrodes. The aim was to place the electrodes in the semicircular canal ampullae. Surgical strategies were developed to deal with the soft tissue obstructions. These procedures were evaluated intraoperatively with microscopic visualization, postoperatively with CT imaging.

**Results:**

The three included patients suffered from bilateral vestibulopathy and hearing loss due to autosomal dominant nonsyndromic sensorineural deafness 9 (DFNA9). A soft tissue obstruction was predicted in one semicircular canal (2 patients) or two semicircular canals (1 patient), based on preoperative imaging. Intraoperatively, bluelining the semicircular canals aided in identifying these locations, by revealing a “whiteline” instead of blueline. Depending on the nature and location of the obstruction, different surgical procedures were employed to facilitate proper electrode insertion. These were as follows: a dummy electrode was used to probe the soft tissue, the obstructive tissue was removed, and/or a bypass fenestration was created. In all patients, the electrodes could be implanted in the semicircular canal ampullae. Based on these first experiences, a diagnostic and surgical guide to deal with obstructions of the semicircular canals during vestibular implantation was developed.

**Conclusions:**

Preoperative imaging can indicate locations of obstructions in the SCCs. Different surgical procedures can be applied to enable appropriate electrode positioning in the SCC ampulla. This article describes the first experiences with obstructions of the semicircular canals during intralabyrinthine vestibular implantation and presents a diagnostic and surgical guide.

**Trial registration:**

ABR NL73492.068.20, METC20-087 (Maastricht University Medical Center) and NAC 11-080 (Geneva University Hospitals).

## Graphical abstract

**Graphical Abstract:**
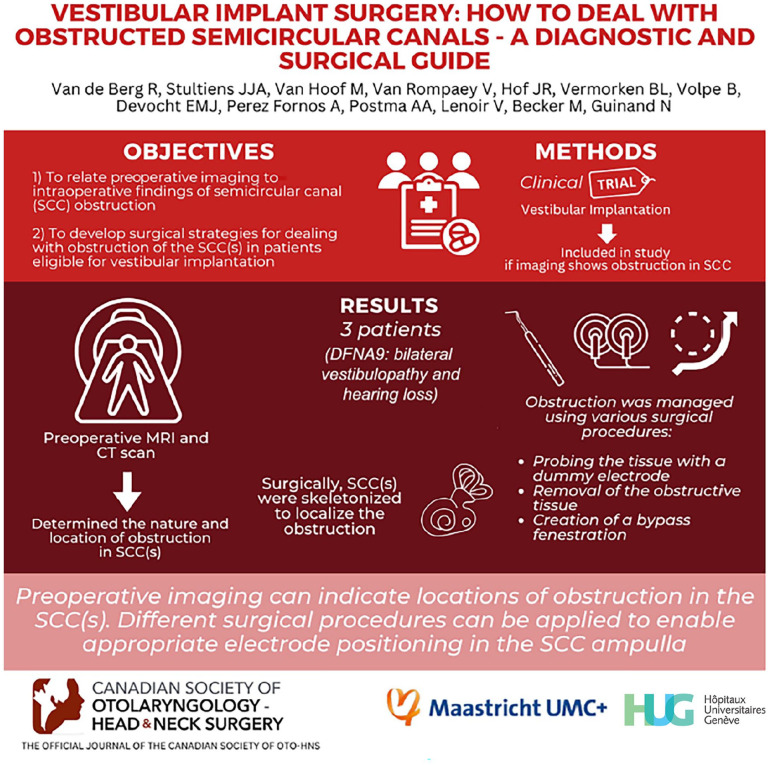

## Introduction

The vestibular implant was developed to treat disabling loss of vestibular function. This prototype artificial balance organ, in concept analogous to a cochlear implant, captures head motion and processes it into electrical signals. These electrical signals are delivered to the vestibular nerve by surgically implanted electrodes. The combined vestibulocochlear implant can partially restore both vestibular function and hearing at the same time.^1^

In recent years, research groups around the world implanted human patients with various prototypes of vestibular and vestibulocochlear implants.^2-5^ A vestibular implant is expected to be feasible as a clinical device in the near future, since a vestibular implant is able to: (partially) restore vestibular mediated reflexes such as the vestibulo-ocular reflex and vestibulo-collic reflex; restore dynamic visual acuity while walking; and influence posture and gait.^6-9^ Furthermore, quality of life tended to improve up to 1 year after implantation, when evaluated in a home use trial.^10^

Two surgical approaches have been described for stimulation of the semicircular canal afferents: the intralabyrinthine approach and the extralabyrinthine approach. For the intralabyrinthine approach, the inner ear is fenestrated and electrodes are inserted in the semicircular canals and placed close to the sensory epithelium.^11^ For the extralabyrinthine approach, the inner ear is not opened and the electrodes are placed near the vestibular ampullary nerve branches close to the semicircular canals.^12,13^ Currently, the intralabyrinthine approach is favored, due to its lower complexity compared with the extralabyrinthine approach, particularly concerning the risks of facial nerve damage and the inability to reach the ampullary nerve canals. One of the main challenges of vestibular implantation using the intralabyrinthine approach is preservation of hearing, as patients are at risk of acquiring a mild high-frequency hearing loss or a profound hearing loss across all frequencies in the implanted ear.^10,14^

Another challenge of the intralabyrinthine approach is the possible presence of obstructions of the labyrinth, which can be quite common in some disorders that lead to loss of vestibular function, such as meningitis, temporal bone trauma, otosclerosis, or genetic disorders such as autosomal dominant nonsyndromic sensorineural deafness 9 (DFNA9; a hereditary loss of hearing and vestibular function due to a mutation in the COCH-gene).^15,16^ Obstructions of the semicircular canals, such as protein deposits, fibrosis, and ossification, could compromise insertion and prevent effective stimulation, as this blocks the way from the predetermined fenestration site toward the sensory epithelium in the ampulla. On the other hand, when the fenestration site is chosen at the ampulla itself, more inner ear damage might be induced and electrode placement may be complicated. Hence, potential obstructions of the labyrinth should first be identified with an adequate preoperative imaging assessment (eg, MRI and CT).

Procedures to handle fibrous tissue or ossification have been well described for cochlear implantation, such as the removal of fibrous tissue at the basal turn, a cochlear drill-out, or the use of double-split electrodes to achieve a higher number of intracochlear stimulus contacts.^16-18^ However, in cochlear implantation the target afferent nerve fibers are located along the whole cochlea, while for vestibular implantation these nerve fibers are only located at a few specific spots. Consequently, reaching these few targets seems important for effective stimulation, and missing one of the targets might significantly influence the clinical result.^19^ The applicability of the types of procedures used for cochlear obstructions is not yet known for vestibular implantation. For these reasons, patients without a patent labyrinth (eg, labyrinthitis ossificans) are not yet considered for intralabyrinthine vestibular electrode insertion in research setting.^20^ However, a subset of patients eligible for vestibular implantation exhibit evidence of a partially patent labyrinth, especially in the semicircular canals, caused by processes such as intralabyrinthine tissue deposits, fibrosis, or ossification.^21-23^ If the semicircular canal ampulla is still patent, or made patent, these patients may still benefit from a vestibular implant. After all, in these cases the target for electrical stimulation is still present. It is therefore imperative to develop procedures to deal with obstructions during vestibular implantation. Cases with a partially-patent labyrinth can enable the development of such procedures for both partially patent and fully obstructed labyrinths.

The objective of this study was to relate preoperative imaging assessment of obstructed semicircular canals to intraoperative findings and to develop a diagnostic and surgical guide that can be used to deal with obstructions of the semicircular canals during intralabyrinthine vestibular implantation.

## Materials and Methods

### Patients

Patients were selected from an ongoing clinical trial evaluating vestibular implantation (ClinicalTrials.gov ID NCT04918745). All patients fitted the diagnostic criteria for bilateral vestibulopathy of the Bárány Society and the vestibular implantation criteria for research, as previously described.^20,24^ Patients were included in the current study when preoperative imaging indicated an obstruction in the semicircular canal. Patients with obstructions in the ampullary end of the canal were excluded for implantation.^20^

### Imaging Assessment

All patients underwent a preoperative high-resolution (HR) CT scan and MRI scan of the temporal bone. For the CT scan, the voxel size was 0.4 mm, and for the HR 3D T2-weighted sequence (1.5 or 3T MRI-scan), this was 0.5 mm. The images were reviewed by experienced radiologists and surgeons to rule out other pathologies and to evaluate obstructions of the labyrinth, in particular of the semicircular canals and cochlea. Postoperatively, another CT scan was made and the positions of the electrodes were determined, using the open source software 3D Slicer (version 5.2).

### The Vestibular Implant and the Surgical Approach

The vestibulocochlear implant design (MED-EL, Innsbruck, Austria), was based on a modified MED-EL Synchrony cochlear implant. It contained 3 vestibular electrode branches with 1 electrode contact for implantation in each semicircular canal and 1 cochlear electrode branch with 9 electrode contacts for cochlear implantation.

Surgeries were performed by surgeons in Maastricht (R.v.d.B.) and Geneva (N.G.). The intralabyrinthine approach was used for semicircular canal implantation, as previously described.^11^ Briefly, a mastoidectomy, facial recess approach, and drilling of the bony overhang of the round window was performed to facilitate cochlear implantation. In addition, the semicircular canals were skeletonized (“bluelined”) to visualize the trajectories of the fluid-filled canal. Next, small fenestrations were made to enable tight fixation of the electrodes in the canals, thus preventing electrode migration. The intended fenestration sites were determined in previous pilot trials of the Geneva-Maastricht team and were described earlier.^25^ The cochlear electrodes were first inserted and then the vestibular electrodes in the patent semicircular canals. In case a semicircular canal obstruction was encountered, different adaptations to the surgical procedure were applied, as presented in the case descriptions below.

### Ethical Considerations

The current results are part of a prospective trial evaluating different aspects of vestibular implantation (ClinicalTrials.gov ID NCT04918745). These results are published early to share the valuable aspects of the preoperative and intraoperative experiences of dealing with obstructions of the semicircular canals. This study was in accordance with the Declaration of Helsinki (amended version 2013). Approval was obtained from the medical ethical committees of Maastricht University Medical Center (ABR NL73492.068.20, METC20-087) and Geneva University Hospitals (NAC 11-080). All participants provided written informed consent.

## Results

### Patient Characteristics

Table 1 presents the characteristics of the 3 included patients. The etiology of their bilateral vestibulopathy was DFNA9. Preoperative imaging suggested obstructions by soft tissue, with or without accompanying calcifications in the labyrinth. In a total of 4 canals, occlusions that compromised (or possibly compromised) electrode insertion were found intraoperatively, in line with the preoperative imaging: twice the superior semicircular canal and twice the posterior semicircular canal. There were no cases in which an obstruction was found intraoperatively that was not detected on preoperative imaging.

**Table 1.** Patient Characteristics.

Case	Sex	Age	Etiology	Implanted ear	Obstructed semicircular canal(s) on implanted side	Surgical site
1	Female	54 y	DFNA9	Right ear	Superior	Maastricht
2	Male	53 y	DFNA9	Left ear	Superior + Posterior	Maastricht
3	Female	67 y	DFNA9	Right ear	Posterior	Geneva

Abbreviation: DFNA9, autosomal dominant nonsyndromic sensorineural deafness 9.

### Case 1: Soft Tissue Obstruction of the Superior Semicircular Canal

#### Preoperative imaging assessment of the labyrinth on the implanted side

**Figure 1.**
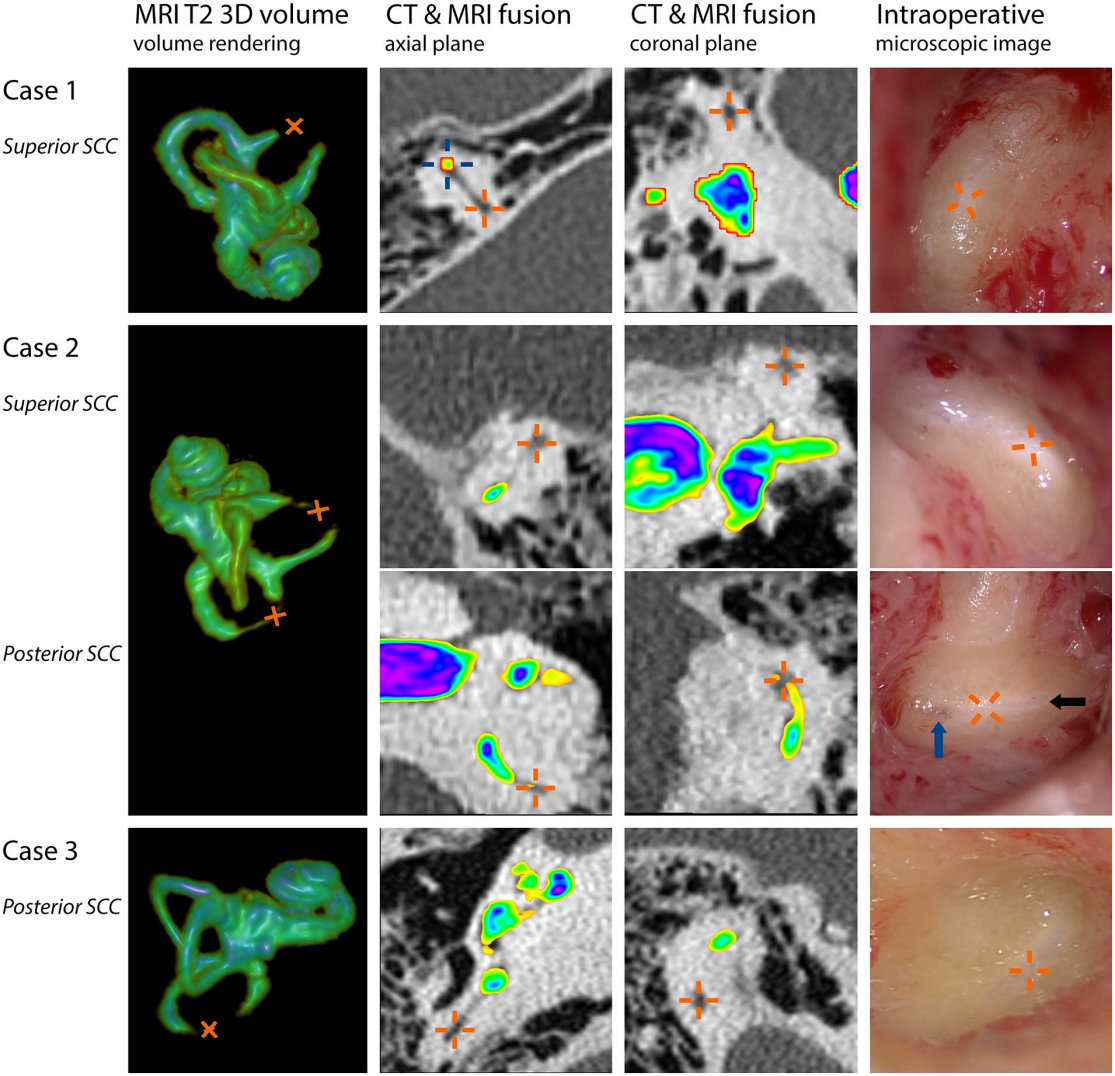
Preoperative MRI and CT findings were compared with intraoperative findings during vestibular implant surgery. Each row represents 1 obstructed semicircular canal from the 3 patients and contains (from left to right): 3-dimensional volume rendering of the inner ear based on T2-weighted MR images, fused images of CT (original shades of gray) and MRI scans (color based on signal intensity) in the axial and coronal planes, and an intraoperative microscopic image after bluelining. Orange crosses represent the locations of obstructive tissue. The fused images here clearly illustrate the suggested patency on CT (low-intensity signal) and obstruction on MRI (absent high-intensity T2 signal). For reference, a blue cross is shown at a patent part of the canal in the axial view of case 1. The different colors of the MRI overlay highlight the subtle T2 signal intensity differences in the canals within the cases. The locations of the absent or noticeably decreased fluid signals on MRI closely corresponded with the locations of the intraoperatively observed soft tissue obstruction of the semicircular canals. In all cases, the areas with obstructive soft tissue could be identified as a white part of the blueline: a “whiteline”. In case of a partial loss of fluid signal on MRI (eg, case 2 posterior canal), intraoperative findings suggested that soft tissue was limited to the edges of the canal walls, resulting in a very small lumen of the canal (black arrow indicating the remaining lumen). Closer to the posterior canal ampulla, the blueline became wider again, suggesting the absence of obstructive tissue (blue arrow). Note that the colors of MR images (signal intensity) cannot be compared between subjects. Image reconstructions were made using the open source software 3D Slicer.

The preoperative CT scan did not show any significant abnormalities. However, the MRI scan (HR T2-weighted images) demonstrated an absence of high-intensity signal around the apex of the superior canal, indicating a discontinuity in fluid. A partial loss of high signal was found for the whole lateral canal, but only around the circumference, which would not cause an obstruction. The posterior canal did not show any significant abnormalities (Figure 1).

#### Intraoperative surgical findings (see Supplemental Material 1, a summarized video recording of the surgical procedure)

**Figure 2.**
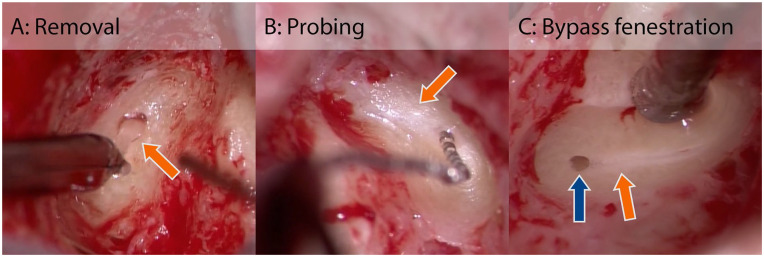
Surgical procedures used to deal with soft tissue obstruction (orange arrows) of the semicircular canals during intralabyrinthine vestibular implantation, as described in this study: (A) Removal of the soft tissue; (B) Probing the canal with a dummy electrode to dilate or mobilize the soft tissue. A dummy electrode can also be used to check the patency of the canal, to prevent damage to the vestibular electrodes; (C) A “bypass fenestration” (blue arrow), between the area with obstruction and the ampulla, to bypass the obstruction.

While bluelining the superior semicircular canal, it was observed that the created blueline could not be extended around the apex of the superior semicircular canal. At this location, instead of the blueline, an opaque white line (from now on referred to as “whiteline”) appeared. While the blueline suggests a clear liquid in the canal, the whiteline suggests dense tissue shining through the meticulously thinned semicircular canal bone. The superior canal was fenestrated and a blockage of the canal was found, which prevented electrode insertion close to the ampullary nerve. The location of the blockage closely corresponded with the absent fluid signal on the MRI scan (Figure 1, orange crosses in upper row). The partially inserted electrode was removed from the canal, and the fenestration was slightly enlarged toward the ampulla. Care was taken to keep the fenestration small to ensure tight electrode fixation after reinsertion. As the blockage consisted of soft tissue, it was possible to remove this using a pointed needle and a microforceps (Figure 2A). After this procedure, it was possible to insert the electrode near the ampullary nerve of the superior canal. Electrode insertion of the other canals was performed without any abnormalities.

#### Postoperative findings

A postoperative CT scan demonstrated that the electrode was successfully implanted in the ampulla of the superior semicircular canal. It was a functional electrode, able to elicit primarily vertically aligned eye movements in response to electrical stimulation and perceptual responses.

### Case 2: Soft Tissue Obstructions of the Superior and Posterior Semicircular Canals

#### Preoperative imaging assessment of the labyrinth on the implanted side

The preoperative CT scan showed a small white density within the apex of the superior semicircular canal. No significant abnormalities were found in the other canals. The MRI scan (HR T2-weighted images) demonstrated an absent fluid signal around the apex of the superior canal and a very thin (approximately 0.2 mm circumference) and hypointense signal halfway the posterior canal (Figure 1). Furthermore, a hypointense signal was observed in the whole lateral canal (visible in Figure 1’s coronal plane of the superior canal, also showing part of the lateral canal: The hypointense signal can be seen in green, compared with the high intensity signal in purple or blue).

#### Intraoperative surgical findings (see Supplemental Material 2, a summarized video recording of the surgical procedure)

After bluelining the superior semicircular canal, the blueline was replaced by a “whiteline” around the apex of the superior semicircular canal, congruent with MRI findings (Figure 1). A fenestration was made around the apex, and removal of the soft tissue was attempted with a pointed needle and microhook. After the removal of some of the soft tissue in the canal, a silicone cochlear implant dummy electrode with a diameter of 0.6 mm at the base tapered to 0.4 mm at the tip was used to probe the canal to check the patency of the canal and as an attempt to dilate or mobilize the tissue (Figure 2B). It was found that the canal was not patent enough for electrode insertion. Instead of further removal (which would require an unwanted increase in the bony fenestration diameter, larger than in case 1), it was decided to drill a “bypass fenestration” anteriorly, between the ampulla and the area with the obstructive tissue, to bypass this area. The dummy electrode was used again, which confirmed the patency of the canal from the bypass fenestration to the ampulla. Bluelining of the posterior canal suggested a narrow lumen: The blueline became very thin in the area around the intersection of the canal with Donaldson’s line, with a partial “whiteline” around its borders (Figure 1, black arrow). Closer to the ampulla, the blueline became wider again, suggesting a completely fluid-filled canal at that location (Figure 1, blue arrow). It was therefore decided to drill a bypass fenestration at that specific location between the ampulla and the obstructed area (Figure 2C), instead of the initially desired “standard” fenestration site around Donaldson’s line. After the abovementioned procedures, vestibular electrode insertion of all canals was performed without any additional abnormalities.

#### Postoperative findings

A postoperative CT scan demonstrated that both electrodes were successfully implanted in the ampullae of the superior and posterior semicircular canals. Both electrodes were able to electrically elicit perceptual responses, but no eye movements. This was also the case for the electrode implanted in the lateral (unobstructed) semicircular canal.

### Case 3: Soft Tissue Obstruction of the Posterior Semicircular Canal

#### Preoperative imaging assessment of the labyrinth on the implanted side

The preoperative CT scan showed no abnormalities. The preoperative MRI scan of the right inner ear revealed an absence of fluid signal in the postero-inferior part of the posterior semicircular canal. The remaining part of the same canal, as well as the superior and lateral semicircular canals, displayed a normal fluid signal (Figure 1). As the preoperative CT scan showed no ossification, the diagnosis of soft tissue obstruction was made.

#### Intraoperative surgical findings (see Supplemental Material 3, a summarized video recording of the surgical procedure)

After bluelining the semicircular canals, a “whiteline” of the posterior semicircular canal was found, congruent with MRI findings (Figure 1). Opening of the posterior semicircular canal confirmed the presence of obstructive soft tissue. After additional drilling and opening of the posterior canal toward the ampulla, a hook was used to remove the residual tissue and to finalize the access to the patent part of the posterior canal. A dummy electrode confirmed the patency of the canal. The implant electrode was then inserted without resistance. Eventually, electrodes could be inserted in all semicircular canals without additional abnormalities.

#### Postoperative findings

A postoperative CT scan demonstrated that the electrode was successfully implanted in the ampulla of the posterior semicircular canal. It was functional, able to electrically elicit a mainly vertically aligned vestibulo-ocular reflex and perceptual responses.

## Discussion

This article presents the relationship between preoperative imaging assessment and intraoperative findings of semicircular canal obstructions during vestibular implantation. In addition, it describes surgical procedures to deal with obstructions of the semicircular canals in patients eligible for vestibulocochlear implantation. Mastering the solutions to these semicircular canal obstructions is key to successful vestibular implant surgery. Here, we describe the first experiences and summarize them in a diagnostic and surgical guide.

The three main findings are as follows: (1) Absent fluid signals on T2-weighted MRI may correspond with the area of obstruction found intraoperatively; (2) Meticulous bluelining of the canals can help to identify the area of soft tissue obstruction, observed as a white line; (3) Surgical procedures to deal with obstruction may include removal, probing with a dummy electrode or a hook, and using a “bypass fenestration” between the ampulla and the obstructed area.

Current challenges for vestibular implantation were recently described.^26^ Proper electrode positioning seems to be crucial for effective electrical stimulation of the ampullary nerve afferents. As presented in this study, obstructions in the semicircular canals could have compromised electrode insertion, but these were successfully managed. It is imperative to investigate multiple strategies to deal with various obstructions of the semicircular canals during intralabyrinthine vestibular implantation. Based on the current experiences, a preliminary proposal for approaching these surgeries will be discussed below.

### Step 1: Preoperative Imaging

Preoperative imaging can be considered as the first step to assess the patency of the semicircular canals. This study showed that hypointense areas due to absent fluid signals on high-resolution T2-weighted MR images can precisely reveal the areas of soft tissue obstruction in the semicircular canals. Certain etiologies of vestibulopathy can show a gradual development of labyrinthine fibrosis, calcification, and later ossification. On CT, semicircular canal obstruction such as fibrosis can be entirely missed unless there is associated calcification/ossification. However, on MRI, fibrosis typically presents as an area of low signal intensity on T2- and T1- weighted MR images.^27^ After intravenous administration of gadolinium, early fibrosis tends to enhance on T1-weighted images because of the ongoing inflammation with increased vascularization, whereas long standing fibrosis typically does not enhance as it mainly consists of scar tissue almost exclusively containing collagen bundles.^28^ In the long run, fibrosis may also show gradual calcification or ossification. As calcification occurs only at a later stage, MRI is superior to CT for the assessment of fibrosis. Nevertheless, MRI and CT are complementary as both ossification/calcification and fibrosis may display a low signal intensity on T2-weighted images. It is the combination of a normal aspect of the labyrinth on CT (ie, lack of ossification) and absent fluid signal on MRI that enables distinction between fibrosis and ossification.^27^ Therefore, whenever intralabyrinthine vestibular implantation is considered, it could be valuable to perform a CT scan as well as an MRI scan with at least HR T2-weighted images.

### Step 2: Surgical Planning

After the assessment of preoperative imaging, a decision should be made on how to surgically deal with the semicircular canal obstruction. This depends on the preferred fenestration sites, the method of electrode fixation, and the preferred surgical approach. In case no fenestration site is preferred, the obstruction could easily be bypassed by fenestrating the canals near their ampullary ends. However, some factors could argue against this location. Drilling close to the ampulla might damage the neural structures and might have a higher risk of hearing loss in patients with residual hearing.^29^ Additionally, extensive manipulation close to the ampulla and accumulated bone dust in the ampulla might impact conductivity postoperatively. Furthermore, inserting the electrodes close to the ampulla could lead to suboptimal electrode positioning. This latter refers to a previous temporal bone pilot trial of the Geneva-Maastricht team, which indicated that the inserted part of the electrode should ideally cover at least a certain distance (approximately one-third of the length of the canal), to facilitate better electrode fixation and positioning. Regarding electrode fixation, the current electrode fixation strategy also involves drilling small fenestrations, to enable tight fixation of the electrodes. This limits electrode migration per- and postoperatively. In case tight fixations are not required, fenestrations could be widened to ease the removal of obstructive soft tissue. Additional fascia around the electrodes could close the fenestrations and fixate them. In situations of extreme fibrosis, the whole canal could be opened up to the ampulla and the canal could be closed again with, for example, cartilage, fascia and/or bone pate, similar to labyrinthine fistulas.^30^ However, this might possibly also impact residual hearing when present. The Geneva-Maastricht team did not use the latter options (yet) since the healing process cannot be well controlled, and this approach might lead to electrode migration postoperatively. Regarding the surgical approach, the intralabyrinthine approach is currently preferred. However, in the case of severe fibrosis, the extralabyrinthine approach might be a useful alternative. The advantage of the extralabyrinthine approach is that the inner ear is not opened. Consequently, intralabyrinthine obstruction does not impede vestibular implantation.

### Step 3: Surgery

In addition to the considerations mentioned above, special concern should be taken during bluelining of the semicircular canals, as this can help to identify areas of obstructive tissue in the semicircular canal. After intraoperative identification of the semicircular canal obstructions, a decision can be made to pursue the planned strategy or to deviate from it, to ensure proper electrode placement. The surgical management procedures are discussed below.

### Overall Management Strategy for Obstructions—Diagnostic and Surgical Guide

**Figure 3.**
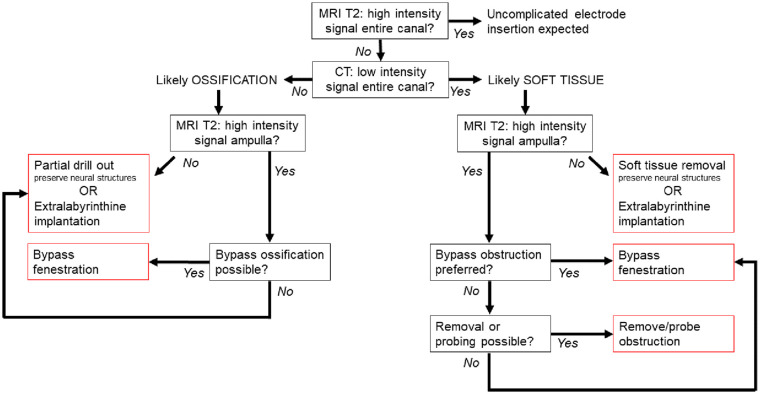
Flowchart of the proposed diagnostic and surgical guide to deal with soft tissue obstruction and ossification of the semicircular canals in patients undergoing vestibular implantation surgery.

Taking the abovementioned considerations and/or limitations into account for the intralabyrinthine approach, a strategy could be proposed to deal with obstructions of the semicircular canals selected for vestibular implantation. Ossification is included in this proposal, since it also comprises electrode insertion by blocking the semicircular canals, albeit with different properties (eg, more firm and fixed). Figure 3 presents the flowchart of this strategy. In case obstruction of a semicircular canal is expected from the imaging, it is preferred to create a bypass fenestration if the obstruction is far away from the ampulla (eg, >⅓ of the length of the canal) and/or when residual hearing (not eligible for cochlear implantation) is present in the ear to be implanted. After all, manipulation of soft tissue could damage the endolymphatic compartment, which might lead to hearing loss. If soft tissue obstruction is present close to the ampulla, removal should be attempted in patients eligible for vestibulocochlear implantation. The removal of the obstruction allows for a fenestration site more far away from the ampulla, which might facilitate better positioning of the electrodes (see above). Removal can be attempted with a pointed needle or a microhook, but other instruments such as endodontic files can also be considered. Furthermore, probing with a dummy electrode may be performed to mobilize the soft tissue to facilitate removal, or to dilate the soft tissue within the canal. Care should be taken to avoid mobilizing the tissue to a difficult to reach location very close to the ampulla, as this complicates the procedure even further. It is currently unknown whether unremoved mobilized soft tissue (eg, toward the ampulla and sensory epithelium) affects vestibular stimulation efficacy. Probing (eg, for dilating) might also be an alternative in patients with residual hearing, since only probing the semicircular canals with a dummy electrode does not immediately lead to a severe decline in hearing, as was measured with auditory brainstem responses (Stultiens et al, manuscript in preparation). An obstruction in the ampulla/region of the crista is currently an exclusion criterion for vestibular implantation, as removal of tissue from the ampulla could damage its neural structures, which might lead to less effective vestibular stimulation. This risk was deemed lower for the removal of tissue in only nonampullary regions. This criterion is also in accordance with the previously published opinion statement on vestibular implantation criteria for research.^20^ However, in the future, it could be considered to attempt removal with preservation of the neural structures as much as possible. Alternatively, the extralabyrinthine approach could be used.

In case ossification of a semicircular canal is likely (canal not patent on both CT and MRI), currently a bypass fenestration would be preferred in all cases with a patent ampulla, since extensive drilling needed to deal with the ossified structures might compromise electrode stability after insertion, as well as residual hearing (if present). An ossified ampulla is currently also an exclusion criterion for vestibular implantation. However, in the future a (partial) drill out with an attempt to preserve neural structures (eg, preserve the bony ridge where the nerves are located^31^) could be considered, in addition to the extralabyrinthine approach.

### Analysis of Tissue Deposits in DFNA9

It is currently unknown whether the presence of tissue deposits in a semicircular canal is a prognostic indicator of less effective electrical vestibular stimulation. In one of the presented cases, electrical stimulation in the ampulla could not elicit clear eye movements (in contrast to perception). However, this was observed in all semicircular canals, both obstructed and unobstructed. His vestibulopathy resulted from DFNA9, a heterogenous disease due to a COCH-gene mutation, which might also lead to deposits in the vestibular nerve area, atrophy of the ampullary nerve, and loss of Scarpa’s ganglion cells.^23,32,33^ The extend of the disease might have contributed to the heterogeneity in eye responses.

The tissue deposits are likely related to aggregation of cochlin, an extracellular matrix protein that is found in the cochlea and the vestibular organ, which is affected by DFNA9 mutations.^23^ Although not the primary aim of this study, samples of the obstructive tissue were intraoperatively collected in 2 of the patients (cases 1 and 2) and an exploratory analysis was performed. Previous (postmortem) light-microscopic investigations showed that these intralabyrinthine deposits in patients with DFNA9 were eosinophilic, acellular, homogeneous and also contained a mucopolysaccharide-like substance.^34^ Electron microscopy showed a highly branched, disarrayed, microfibrillar substance, along with scattered glycosaminoglycan-like granules.^35^ Immunostaining indicated that extracellular cochlin is present in this deposition.^23^ As was shown here, by removing the obstructions, the opportunity to investigate the unresolved pathological process in vivo becomes possible. Our analysis using conventional histopathology, two-photon microscopy, ultraviolet and visible spectroscopy, and protein electrophoresis yielded no new leads (not presented) in further discriminating the contents or structure of the obstruction. Considering the previously found excess of microfibrillar substance and type II collagen degradation,^35^ future investigations could focus on reconstructing retrieved aggregates in 3D using electron microscopy,^36^ to further clarify this relationship.

### Future

This article describes the first experiences with obstructions in the semicircular canals during intralabyrinthine vestibular implantation. Based on these experiences with preoperative imaging assessment, intraoperative findings, and applied surgical procedures, a diagnostic and surgical guide was developed. As the number of vestibular implantations increases, more experience will increase understanding on how to deal with obstructions of the canals. Therefore, the discussed management strategy is a preliminary proposal, which is not set in stone. Furthermore, the presence of obstructions in the labyrinth might become one of the parameters to determine the side to be implanted.

## Conclusions

Obstructions of the semicircular canals can be encountered during intralabyrinthine vestibular implantation. In these cases, preoperative imaging may foresee intraoperative findings, and meticulous bluelining of the semicircular canals can help to identify the area of obstruction. Different surgical procedures such as removing, probing, and bypassing the obstruction can be used to facilitate proper electrode positioning. The developed surgical and diagnostic guide can be used to deal with obstructions of the semicircular canals in vestibular implantation.
